# Supplementary material for: The Small RNA Universe of Capitella teleta
Source: Front Mol Biosci. 2022 Feb 25;9:802814. doi: 10.3389/fmolb.2022.802814 (PMC8915122; doi:10.3389/fmolb.2022.802814)
Supplement: Supplementary file 1 [file DataSheet1.ZIP › Supplement/known/CAPTEscaffold_291_16152.pdf]

Provisional ID	:	CAPTEscaffold_291_16152
Score total	:	9889.6
Score for star read(s)	:	3.9
Score for read counts	:	9879
Score for mfe	:	2.1
Score for randfold	:	1.6
Score for cons. seed	:	3
Total read count	:	19389
Mature read count	:	18838
Loop read count	:	0
Star read count	:	551

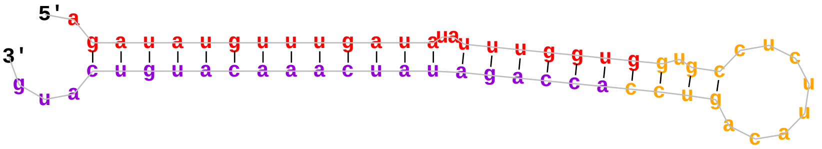

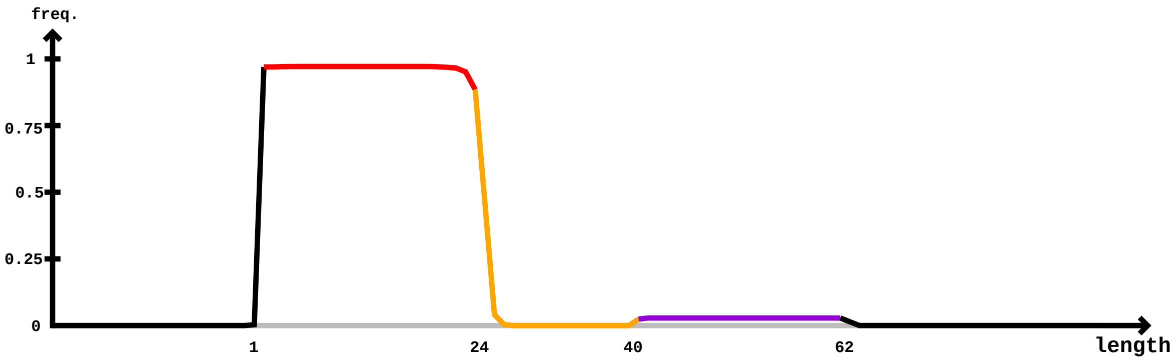

Mature Star

```
5'- cccacugccuccucucggcaagauauguuugauauauuuggugggugccucuuacaguccaccagauaucaaacaugucauguggaggguggcuguacacuggauguaacagc   -3'  obs
    cccacugccuccucucggcaagauauguuugauauauuuggugggugccucuuacaguccaccagauaucaaacaugucauguggaggguggcuguacacuggauguaacagc        exp
    cccacugccuccucucggcaagauauguuugauauauuuggugggugccucuuacaguccaccagauaucaaacaugucauguggaggguggcuguacacuggauguaacagc        known
    ...((.(((.((.(((.(((.((((((((((((..((((((((.((........))))))))))))))))))))))).))).))))).))).))...(((.......))).   reads   mm   sample

    ...................aagauauguuugauauauuuggu......................................................................   9       0    seq
    ...................aGgauauguuugauauauuuggu......................................................................   5       1    seq
    ...................aagauauguuugauauauuuggug.....................................................................   41      0    seq
    ...................aGgauauguuugauauauuuggug.....................................................................   6       1    seq
    ...................aGgauauguuugauauauuuggugg....................................................................   1       1    seq
    ....................Ggauauguuugauauauu..........................................................................   1       1    seq
    ....................agauauguuugauauauu..........................................................................   14      0    seq
    ....................agauauguuugauauauuu.........................................................................   40      0    seq
    ....................agauauguuugauauauuAg........................................................................   1       1    seq
    ....................agauauguuugauauauuug........................................................................   52      0    seq
    ....................agauauguuugauauauuugg.......................................................................   269     0    seq
    ....................agaAauguuugauauauuugg.......................................................................   1       1    seq
    ....................Ggauauguuugauauauuugg.......................................................................   1       1    seq
    ....................Ngauauguuugauauauuugg.......................................................................   1       1    seq
    ....................agauauguuugauauauuugA.......................................................................   2       1    seq
    ....................agauauguuugauauauuuAg.......................................................................   1       1    seq
    ....................agauauguuugauauauuugAu......................................................................   1       1    seq
    ....................agauaugAuugauauauuuggu......................................................................   1       1    seq
    ....................agaGauguuugauauauuuggu......................................................................   2       1    seq
    ....................agauauguuugauauauuCggu......................................................................   1       1    seq
    ....................agauauguuugauauauuuAgu......................................................................   1       1    seq
    ....................agauauguuugauauauuuggA......................................................................   15      1    seq
    ....................agauauguuugauauauuuggG......................................................................   1       1    seq
    ....................agauaAguuugauauauuuggu......................................................................   1       1    seq
    ....................agauaugGuugauauauuuggu......................................................................   1       1    seq
    ....................agauauguuugauauauuuggu......................................................................   1247    0    seq
    ....................agauauguuugauauGuuuggu......................................................................   1       1    seq
    ....................agauauguuugauaAauuuggu......................................................................   2       1    seq
    ....................Ggauauguuugauauauuuggu......................................................................   6       1    seq
    ....................aAauauguuugauauauuuggu......................................................................   1       1    seq
    ....................agauauguuugauauauuuggC......................................................................   1       1    seq
```

Mature Star

```
cccacugccuccucucggcaagauauguuugauauauuuggugugccucuuacaguccaccagauaucaaacaugucauguggaggguggcuguacacuggauguaacagc
....................aUauauguuugauauauuuggug.....................................................................	2	1	seq
....................agauauAuuugauauauuuggug.....................................................................	1	1	seq
....................aNauauguuugauauauuuggug.....................................................................	1	1	seq
....................agauauguuugauauauuuCgug.....................................................................	1	1	seq
....................agauauguuCgauauauuuggug.....................................................................	2	1	seq
....................agauauguuugauauauuuggGg.....................................................................	2	1	seq
....................agaAauguuugauauauuuggug.....................................................................	10	1	seq
....................aAauauguuugauauauuuggug.....................................................................	3	1	seq
....................agauauguCugauauauuuggug.....................................................................	1	1	seq
....................Ngauauguuugauauauuuggug.....................................................................	1	1	seq
....................agauauguuugauauauuugguA.....................................................................	11	1	seq
....................agauauguuugauauaAuuggug.....................................................................	2	1	seq
....................agauauguuugauauaGuuggug.....................................................................	4	1	seq
....................agauauguuugGuauauuuggug.....................................................................	2	1	seq
....................agauauguuuAauauauuuggug.....................................................................	12	1	seq
....................agauauguuugauauauuAggug.....................................................................	2	1	seq
....................agauauguuugauauauuCggug.....................................................................	1	1	seq
....................agauauguuugauauauuuggug.....................................................................	8440	0	seq
....................agauauguuuUauauauuuggug.....................................................................	2	1	seq
....................agauauguuugauauauAuggug.....................................................................	1	1	seq
....................agauauguuugUuauauuuggug.....................................................................	2	1	seq
....................agauauguuGgauauauuuggug.....................................................................	1	1	seq
....................agauauguuugaAauauuuggug.....................................................................	1	1	seq
....................agauauguuugauauauuugUug.....................................................................	1	1	seq
....................agauauguAugauauauuuggug.....................................................................	3	1	seq
....................agauauguuugauauauuugguC.....................................................................	2	1	seq
....................Ggauauguuugauauauuuggug.....................................................................	49	1	seq
....................agUuauguuugauauauuuggug.....................................................................	1	1	seq
....................agauauguuugauauauuuggCg.....................................................................	1	1	seq
....................agauauguuugauauauuuUgug.....................................................................	3	1	seq
....................agauauCuuugauauauuuggug.....................................................................	1	1	seq
....................agaGauguuugauauauuuggug.....................................................................	11	1	seq
....................agauaugCuugauauauuuggug.....................................................................	1	1	seq
....................agauauguuugaGauauuuggug.....................................................................	7	1	seq
....................agauauguuugauaAauuuggug.....................................................................	2	1	seq
....................agauauguuugauauauuugguU.....................................................................	10	1	seq
....................agauauguuAgauauauuuggug.....................................................................	5	1	seq
....................agauaugAuugauauauuuggug.....................................................................	4	1	seq
....................agauauguuugauauauuuggAg.....................................................................	2	1	seq
....................agauauguuugauauauuGggug.....................................................................	1	1	seq
....................agauauguuugauauauuuAgug.....................................................................	21	1	seq
....................agauauguuugauauUuuuggug.....................................................................	1	1	seq
....................agauauguuugauauaCuuggug.....................................................................	1	1	seq
....................agaGauguuugauauauuuggugg....................................................................	6	1	seq
....................agauaAguuugauauauuuggugg....................................................................	4	1	seq
....................agauauguuugauauaAuuggugg....................................................................	3	1	seq
....................agauauguuugauauauuuAgugg....................................................................	12	1	seq
....................agauauguuuUauauauuuggugg....................................................................	1	1	seq
....................agauauguuugauauauuugAugg....................................................................	1	1	seq
....................agauauguuugauauauuCggugg....................................................................	1	1	seq
....................aNauauguuugauauauuuggugg....................................................................	1	1	seq
....................agauaGguuugauauauuuggugg....................................................................	1	1	seq
....................agauauguuugaGauauuuggugg....................................................................	2	1	seq
....................agauauguuugauUuauuuggugg....................................................................	2	1	seq
....................agaAauguuugauauauuuggugg....................................................................	9	1	seq
....................agauauguuugauauauuugUugg....................................................................	3	1	seq
....................agauauguuugauauauuuggAgg....................................................................	1	1	seq
....................agauauguGugauauauuuggugg....................................................................	1	1	seq
....................agauauguuugauGuauuuggugg....................................................................	1	1	seq
....................Cgauauguuugauauauuuggugg....................................................................	1	1	seq
....................agauauguuugauauauuugguCg....................................................................	1	1	seq
....................Ugauauguuugauauauuuggugg....................................................................	2	1	seq
....................agauauguuugauauauuuUgugg....................................................................	1	1	seq
....................agauauguuuAauauauuuggugg....................................................................	7	1	seq
....................agaCauguuugauauauuuggugg....................................................................	1	1	seq
....................agauauguuugauaGauuuggugg....................................................................	2	1	seq
....................agauauguuugauaAauuuggugg....................................................................	3	1	seq
....................agauauguuugauauauuuggugC....................................................................	1	1	seq
....................agauauguuugGuauauuuggugg....................................................................	1	1	seq
....................agauaugCuugauauauuuggugg....................................................................	1	1	seq
```

```
                    Mature                                      Star
cccacugccuccucucggcaagauauguuugauauauuuggugggugccucuuacaguccaccagauaucaaacaugucauguggaggguggcuguacacuggauguaacagc
....................aAauauguuugauauauuuggugg....................................................................  3     1    seq
....................agauauguAugauauauuuggugg....................................................................  3     1    seq
....................agauauguuugaAauauuuggugg....................................................................  1     1    seq
....................agauauguuugauauauuuggCgg....................................................................  2     1    seq
....................agauauguuugauauauuugguUg....................................................................  1     1    seq
....................agauauguuugauauauuuggugA....................................................................  32    1    seq
....................agauauguuAgauauauuuggugg....................................................................  3     1    seq
....................agauauguuugauauauuuggugU....................................................................  122   1    seq
....................Ngauauguuugauauauuuggugg....................................................................  1     1    seq
....................agauauguuugauauauuugguAg....................................................................  11    1    seq
....................Ggauauguuugauauauuuggugg....................................................................  20    1    seq
....................agauauguuugaCauauuuggugg....................................................................  2     1    seq
....................agauauguuugauauauuuggugg....................................................................  7370  0    seq
....................agauauguuugauauauAuggugg....................................................................  2     1    seq
....................agauauguCugauauauuuggugg....................................................................  2     1    seq
....................agauauguuugauauauuAggugg....................................................................  2     1    seq
....................agauaugAuugauauauuuggugg....................................................................  2     1    seq
....................agauauguuugauaCauuuggugg....................................................................  1     1    seq
....................agauauguuugauauauuGgguggu...................................................................  1     1    seq
....................agauauguuugauauauuugguggC...................................................................  7     1    seq
....................agUuauguuugauauauuugguggu...................................................................  1     1    seq
....................agauauguuugauauauuugguggu...................................................................  337   0    seq
....................agauauguuugauauauuugguggA...................................................................  367   1    seq
....................agauaAguuugauauauuugguggu...................................................................  1     1    seq
....................agauauguuugauauauuuAguggu...................................................................  1     1    seq
....................Ggauauguuugauauauuugguggu...................................................................  1     1    seq
....................agauauguuugauauauuugguggG...................................................................  3     1    seq
....................agauauguuugauauauuuggugguU..................................................................  71    1    seq
....................agauauguuugauauauuuggugguA..................................................................  5     1    seq
....................agauauguuugauauauuuggugguC..................................................................  3     1    seq
......................auauguuCgauauauuuggug.....................................................................  1     1    seq
......................auauguuugauauauuuggug.....................................................................  4     0    seq
......................aGauguuugauauauuuggug.....................................................................  2     1    seq
......................aGauguuugauauauuuggugg....................................................................  9     1    seq
......................auauguuugauauauuuggugg....................................................................  4     0    seq
......................aGauguuugauauauuugguggu...................................................................  1     1    seq
.......................uauguuugauauauuugg.......................................................................  5     0    seq
.......................uauguuugauauauuuggu......................................................................  1     0    seq
.......................uauguuugauauauuuggug.....................................................................  1     0    seq
........................auguuugauauauuuggug.....................................................................  2     0    seq
........................auguuugauauauuuggugg....................................................................  1     0    seq
...........................................................accagauaucaaacauguca.................................  1     0    seq
...........................................................accagauaucaaacaugucau................................  2     0    seq
...........................................................accagGuaucaaacaugucaug...............................  9     1    seq
...........................................................accagauaucaaacauguAaug...............................  1     1    seq
...........................................................accagauaucaaGcaugucaug...............................  1     1    seq
...........................................................accagauaucaaacaugucaAg...............................  1     1    seq
...........................................................accaAauaucaaacaugucaug...............................  1     1    seq
...........................................................accagauaucaaacaugucaug...............................  452   0    seq
...........................................................accagauauUaaacaugucaug...............................  1     1    seq
...........................................................Gccagauaucaaacaugucaug...............................  2     1    seq
...........................................................accagaAaucaaacaugucaug...............................  1     1    seq
............................................................ccagauGucaaacaugucaug...............................  1     1    seq
............................................................ccagauaucaaacaugucaug...............................  75    0    seq
............................................................Acagauaucaaacaugucaug...............................  1     1    seq
............................................................ccagauaucaaacaugucaugu..............................  1     0    seq
...............................................................gauaucaaacaugucaug...............................  1     0    seq
.................................................................................uggaggguggcuguacacuggaugua.....  1     0    seq
.................................................................................uggaggguggcuguacacuggauguaa....  1     0    seq
```
